# Dichlorido(4,7-diaza-1-azoniacyclo­nonane-κ^2^ *N* ^4^,*N* ^7^)palladium(II) *p*-toluene­sulfonate

**DOI:** 10.1107/S1600536810016533

**Published:** 2010-05-12

**Authors:** Timothy A. Anthony, Hadi D. Arman, Judith A. Walmsley

**Affiliations:** a Department of Chemistry, The University of Texas at San Antonio, One UTSA Circle, San Antonio, Texas 78249-0698, USA

## Abstract

The title compound, [PdCl_2_(C_6_H_16_N_3_)](C_7_H_7_SO_3_), consists of a Pd^II^ atom bonded to two N atoms of the 1,4,7-triaza­cyclo­nonane (TACN) ligand and two chloride ions, which define a distorted square-planar geometry. The third N atom of the TACN ligand is protonated and hydrogen bonds to the *p*-toluene­sulfonate anion. The Cl—Pd—Cl angle is larger than the N—Pd—N angle. The packing is dominated by layers, which are formed by the criss-crossing of two different hydrogen-bonded chains. One chain is composed of hydrogen-bonded Pd(TACNH)Cl_2_^+^ cations, while the second is formed through hydrogen bonding between the *p*-toluene­sulfonate anion and the Pd(TACNH)Cl_2_^+^ cation.

## Related literature

For background to complexes of Pd^II^ and Pt^II^ with 1,4,7-triaza­cyclo­nonane (TACN), see: McAuley & Whitcombe (1988 ▶); Blake *et al.* (1988 ▶, 1993 ▶); Margulis & Zompa (1992 ▶); Hunter *et al.* (1988 ▶); Davies *et al.* (2000 ▶). For the synthesis of TACN, see: Kang & Jo (2003 ▶). For Pd—N and Pd—Cl bond distances in Pd(en)Cl_2_, see: Iball *et al.* (1975 ▶).
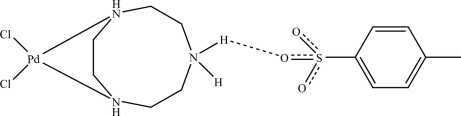


## Experimental

### 

#### Crystal data

[PdCl_2_(C_6_H_16_N_3_)](C_7_H_7_O_3_S)
*M* *_r_* = 478.70
Triclinic, 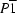

*a* = 6.6663 (11) Å
*b* = 7.0023 (11) Å
*c* = 19.646 (3) Å
α = 92.149 (3)°
β = 92.301 (3)°
γ = 103.084 (4)°
*V* = 891.5 (2) Å^3^
*Z* = 2
Mo *K*α radiation
μ = 1.47 mm^−1^
*T* = 98 K
0.39 × 0.25 × 0.14 mm

#### Data collection

Rigaku AFC12 Kappa goniometer diffractometer
Absorption correction: multi-scan (*ABSCOR*; Higashi, 1995 ▶) *T*_min_ = 0.839, *T*_max_ = 1.000
6224 measured reflections
3998 independent reflections
3941 reflections with *I* > 2σ(*I*)
*R*_int_ = 0.018

#### Refinement

*R*[*F* ^2^ > 2σ(*F* ^2^)] = 0.029
*wR*(*F* ^2^) = 0.067
*S* = 1.00
3998 reflections
220 parameters
H atoms treated by a mixture of independent and constrained refinement
Δρ_max_ = 0.62 e Å^−3^
Δρ_min_ = −0.98 e Å^−3^

### 

Data collection: *CrystalClear* (Rigaku/MSC, 2005 ▶); cell refinement: *CrystalClear*; data reduction: *CrystalClear*; program(s) used to solve structure: *SHELXS97* (Sheldrick, 2008 ▶); program(s) used to refine structure: *SHELXL97* (Sheldrick, 2008 ▶); molecular graphics: *ORTEPII* (Johnson, 1976 ▶); software used to prepare material for publication: *publCIF* (Westrip, 2010 ▶).

## Supplementary Material

Crystal structure: contains datablocks global, I. DOI: 10.1107/S1600536810016533/tk2666sup1.cif

Structure factors: contains datablocks I. DOI: 10.1107/S1600536810016533/tk2666Isup2.hkl

Additional supplementary materials:  crystallographic information; 3D view; checkCIF report

## Figures and Tables

**Table:** 

Pd1—N1	2.030 (2)
Pd1—N2	2.060 (2)
Pd1—Cl1	2.3053 (8)
Pd1—Cl2	2.3115 (7)

**Table:** 

N1—Pd1—N2	82.71 (9)
Cl1—Pd1—Cl2	94.02 (3)

**Table 2:** Hydrogen-bond geometry (Å, °)

*D*—H⋯*A*	*D*—H	H⋯*A*	*D*⋯*A*	*D*—H⋯*A*
N1—H1a⋯O1^i^	0.82 (6)	2.05 (6)	2.833 (3)	159 (6)
N2—H2a⋯Cl1^ii^	0.79 (5)	2.66 (6)	3.365 (2)	149 (5)
N3—H3a⋯O2	0.75 (6)	2.04 (6)	2.743 (3)	156 (6)
N3—H3d⋯Cl2^iii^	0.84 (5)	2.31 (6)	3.136 (3)	171 (5)

Symmetry codes: (i) 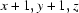; (ii) 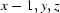; (iii) 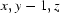.

**Fig. 1.**
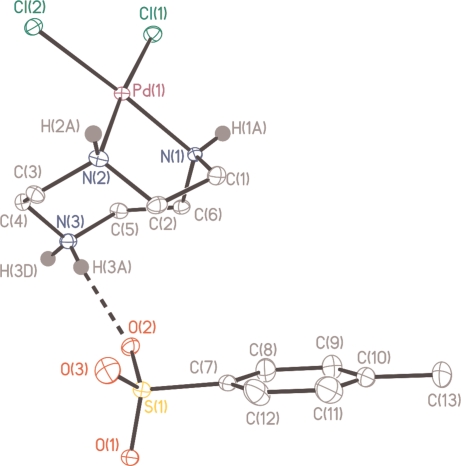
Thermal ellipsoid plot (50% probability) of the asymmetric unit of the title salt. Carbon bound hydrogen atoms are removed for clarity.

**Fig. 2.**
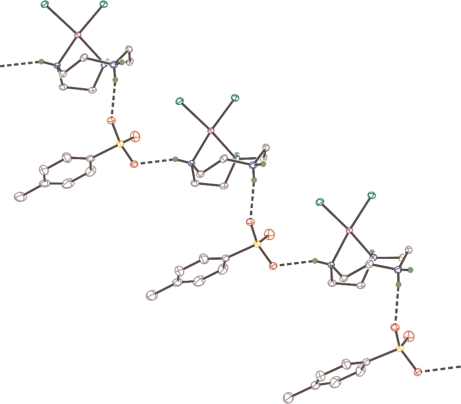
Hydrogen bonded chain formed between the protonated Pd(TACNH)Cl2+ cation and the p-toluenesulfonate anion; non-participating hydrogen atoms have been removed for clarity. Thermal ellipsoids are shown at 50% probability.

**Fig. 3.**
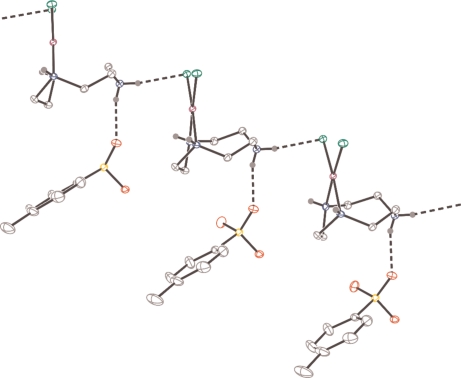
Hydrogen bonded chain formed between protonated Pd(TACNH)Cl2+ ions; non- participating hydrogen atoms have been removed for clarity. Thermal ellipsoids are shown at 50% probability.

**Fig. 4.**
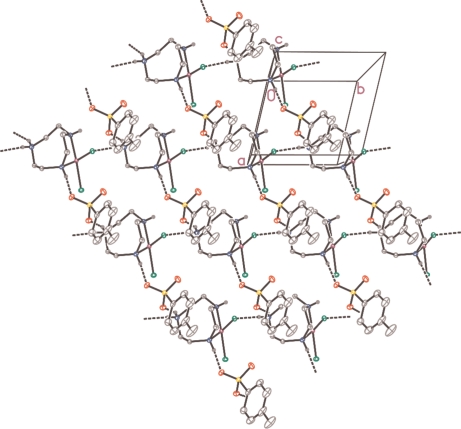
Illustration of the hydrogen bonded layer (view down the c axis); non-participating hydrogen atoms have been removed for clarity. Thermal ellipsoids are shown at 50% probability.

## supplementary crystallographic information

### Comment

Complexes of Pd^II^ and Pt^II^ with 1,4,7,-triazacyclononane (TACN) have been reported in which the metal ion is coordinated to all three of the TACN nitrogen atoms (McAuley & Whitcombe, 1988) or to only two of the N atoms (Blake *et al.*, 1988; Blake *et al.*, 1993; Margulis & Zompa, 1992; Hunter *et al.*, 1988). In the latter case, under acidic conditions, the non-Pd bonded N atom becomes protonated. As a result, hydrogen bonding networks can be formed in the presence of an acceptor site. A similar type of complex has been reported for Pt^II^ (Davies *et al.*, 2000).

The title salt is comprised of a protonated Pd(TACNH)Cl_2_^+^ cation and a *p*-toluenesulfonate ion, Fig. 1. The Pd—N and Pd—Cl bond distances, Table 1, are similar to the bond distances observed in Pd(en)Cl_2_ of 1.9798 (7) and 2.3084 (8) Å for Pd—N and Pd—Cl bonds, respectively (Iball *et al.*, 1975). The geometry about the Pd^II^atom is essentially square planar, but with the Cl1—Pd—Cl2 bond angle larger than the N1—Pd—N2 bond angle., Table 1 The dimer associates into a supramolecular chain *via* a nine member, ···O1—S1—O2···H3a—N3—C—C—N1—H1a···, synthon, Fig 2. A second hydrogen bonded chain is observed that is formed between protonated Pd(TACNH)Cl_2_^+^ cations. These chains are composed of a seven member, ···Cl2—Pd—N1—C—C—N3—H3d···, repeat unit involving the protonated N atom, Fig 3. Hydrogen bonding distances are given in Table 1. These two hydrogen bonded chains are situated approximately perpendicular to one another which allows for the formation of 2-D hydrogen bonded layers, Fig 4.

### Experimental

The ligand (TACN) was prepared according to the procedure reported in the literature (Kang & Jo, 2003). K_2_PdCl_4_ (0.126 g, 0.387 mmol) was dissolved in deionized H_2_O (20 ml) and heated to 343 K. TACN (0.0500 g, 0.387 mmol) was dissolved in 50% 2-propanol/50% water solution (20 ml) and heated to 343 K. The two hot solutions were combined, removed from the heat and allowed to stir for 48 h. Yellow-brown crystals precipitated and were isolated by suction filtration. These were recrystallized from a 50% 2-propanol/50% water mixture to obtain crystals suitable for X-ray analysis.

### Refinement

Carbon-bound H-atoms were placed in calculated positions(C—H 0.93 - 0.97 Å) and were included in the refinement in the riding model approximation with U_iso_(H) set to 1.2-1.5 U_eq_(C). The nitrogen-bound H-atoms were located in a difference Fourier map and were refined with U_iso_(H) = 1.2U_eq_(N).

### Crystal data

**Table:**

[PdCl_2_(C_6_H_16_N_3_)](C_7_H_7_O_3_S)	*Z* = 2
*M**_r_* = 478.70	*F*(000) = 484
Triclinic, *P*1	*D*_x_ = 1.783 Mg m^−^^3^
Hall symbol: -P 1	Mo *K*α radiation, λ = 0.71073 Å
*a* = 6.6663 (11) Å	Cell parameters from 4441 reflections
*b* = 7.0023 (11) Å	θ = 3.0–40.2°
*c* = 19.646 (3) Å	µ = 1.47 mm^−^^1^
α = 92.149 (3)°	*T* = 98 K
β = 92.301 (3)°	Prism, orange
γ = 103.084 (4)°	0.39 × 0.25 × 0.14 mm
*V* = 891.5 (2) Å^3^	

### Data collection

**Table:**

Rigaku AFC12 Kappa goniometer diffractometer	3998 independent reflections
Radiation source: sealed tube	3941 reflections with *I* > 2σ(*I*)
graphite	*R*_int_ = 0.018
Detector resolution: 14.286 pixels mm^-1^	θ_max_ = 27.5°, θ_min_ = 3.0°
ω scans	*h* = −8→8
Absorption correction: multi-scan (*ABSCOR*; Higashi, 1995)	*k* = −9→8
*T*_min_ = 0.839, *T*_max_ = 1.000	*l* = −25→24
6224 measured reflections	

### Refinement

**Table:**

Refinement on *F*^2^	Primary atom site location: structure-invariant direct methods
Least-squares matrix: full	Secondary atom site location: difference Fourier map
*R*[*F*^2^ > 2σ(*F*^2^)] = 0.029	Hydrogen site location: inferred from neighbouring sites
*wR*(*F*^2^) = 0.067	H atoms treated by a mixture of independent and constrained refinement
*S* = 1.00	*w* = 1/[σ^2^(*F*_o_^2^) + (0.007*P*)^2^ + 4.1*P*] where *P* = (*F*_o_^2^ + 2*F*_c_^2^)/3
3998 reflections	(Δ/σ)_max_ = 0.001
220 parameters	Δρ_max_ = 0.62 e Å^−^^3^
0 restraints	Δρ_min_ = −0.98 e Å^−^^3^

### Special details

**Table:**

Geometry. All esds (except the esd in the dihedral angle between two l.s. planes) are estimated using the full covariance matrix. The cell esds are taken into account individually in the estimation of esds in distances, angles and torsion angles; correlations between esds in cell parameters are only used when they are defined by crystal symmetry. An approximate (isotropic) treatment of cell esds is used for estimating esds involving l.s. planes.
Refinement. Refinement of *F*^2^ against ALL reflections. The weighted *R*-factor wR and goodness of fit *S* are based on *F*^2^, conventional *R*-factors *R* are based on *F*, with *F* set to zero for negative *F*^2^. The threshold expression of *F*^2^ > σ(*F*^2^) is used only for calculating *R*-factors(gt) etc. and is not relevant to the choice of reflections for refinement. *R*-factors based on *F*^2^ are statistically about twice as large as those based on *F*, and *R*- factors based on ALL data will be even larger.

### Fractional atomic coordinates and isotropic or equivalent isotropic displacement parameters (Å^2^)

**Table:**

	*x*	*y*	*z*	*U*_iso_*/*U*_eq_	
Pd1	0.97009 (3)	1.13904 (3)	0.126918 (10)	0.00972 (6)	
Cl2	0.84514 (10)	1.25666 (9)	0.02940 (3)	0.01338 (13)	
Cl1	1.31092 (10)	1.25684 (10)	0.10367 (3)	0.01477 (13)	
N1	1.0477 (4)	1.0307 (3)	0.21612 (12)	0.0107 (4)	
N2	0.6749 (3)	1.0232 (3)	0.15540 (12)	0.0119 (4)	
N3	0.8065 (4)	0.6217 (4)	0.12108 (13)	0.0131 (5)	
C5	1.0277 (4)	0.7097 (4)	0.14512 (15)	0.0135 (5)	
H5A	1.0902	0.7978	0.1110	0.016*	
H5B	1.1000	0.6042	0.1465	0.016*	
C4	0.6940 (4)	0.7353 (4)	0.07501 (14)	0.0133 (5)	
H4A	0.6068	0.6428	0.0422	0.016*	
H4B	0.7954	0.8225	0.0497	0.016*	
C6	1.0657 (4)	0.8215 (4)	0.21375 (14)	0.0125 (5)	
H6A	0.9693	0.7511	0.2449	0.015*	
H6B	1.2032	0.8187	0.2311	0.015*	
C3	0.5617 (4)	0.8563 (4)	0.10909 (15)	0.0139 (5)	
H3B	0.4858	0.9082	0.0739	0.017*	
H3C	0.4616	0.7700	0.1352	0.017*	
C2	0.6799 (4)	0.9703 (4)	0.22882 (14)	0.0150 (5)	
H2B	0.6567	0.8290	0.2315	0.018*	
H2C	0.5715	1.0137	0.2522	0.018*	
C1	0.8864 (4)	1.0674 (4)	0.26239 (14)	0.0143 (5)	
H1B	0.8975	1.2073	0.2694	0.017*	
H1C	0.9036	1.0128	0.3063	0.017*	
S1	0.48600 (10)	0.43932 (10)	0.28213 (3)	0.01295 (14)	
O1	0.3964 (3)	0.2362 (3)	0.29720 (10)	0.0153 (4)	
O2	0.6622 (3)	0.4531 (3)	0.23865 (10)	0.0162 (4)	
O3	0.3348 (4)	0.5425 (3)	0.25768 (12)	0.0240 (5)	
C7	0.5938 (5)	0.5596 (4)	0.36084 (15)	0.0168 (6)	
C8	0.7933 (5)	0.5552 (5)	0.38198 (17)	0.0244 (7)	
H8A	0.8725	0.4937	0.3545	0.029*	
C9	0.8743 (6)	0.6442 (5)	0.44501 (18)	0.0316 (8)	
H9A	1.0087	0.6422	0.4590	0.038*	
C12	0.4771 (6)	0.6539 (5)	0.40103 (18)	0.0286 (7)	
H12A	0.3447	0.6602	0.3861	0.034*	
C11	0.5605 (6)	0.7399 (6)	0.4645 (2)	0.0373 (9)	
H11A	0.4811	0.8014	0.4919	0.045*	
C10	0.7584 (6)	0.7355 (5)	0.48716 (17)	0.0329 (9)	
C13	0.8478 (8)	0.8282 (7)	0.55589 (19)	0.0492 (13)	
H13A	0.7474	0.8842	0.5781	0.074*	
H13B	0.9686	0.9293	0.5496	0.074*	
H13C	0.8842	0.7299	0.5836	0.074*	
H1A	1.158 (8)	1.102 (8)	0.230 (3)	0.059*	
H2A	0.624 (8)	1.114 (8)	0.151 (3)	0.059*	
H3A	0.742 (8)	0.592 (8)	0.151 (3)	0.059*	
H3D	0.809 (8)	0.516 (8)	0.100 (3)	0.059*	

### Atomic displacement parameters (Å^2^)

**Table:**

	*U*^11^	*U*^22^	*U*^33^	*U*^12^	*U*^13^	*U*^23^
Pd1	0.00972 (10)	0.00856 (10)	0.01090 (11)	0.00177 (7)	0.00143 (7)	0.00151 (7)
Cl2	0.0159 (3)	0.0127 (3)	0.0122 (3)	0.0043 (2)	0.0012 (2)	0.0019 (2)
Cl1	0.0111 (3)	0.0167 (3)	0.0161 (3)	0.0015 (2)	0.0024 (2)	0.0038 (2)
N1	0.0111 (11)	0.0110 (11)	0.0091 (11)	0.0001 (8)	0.0017 (8)	0.0009 (8)
N2	0.0092 (10)	0.0109 (11)	0.0162 (12)	0.0035 (8)	0.0025 (8)	0.0011 (9)
N3	0.0149 (11)	0.0112 (11)	0.0130 (12)	0.0024 (9)	0.0021 (9)	0.0012 (9)
C5	0.0099 (12)	0.0132 (13)	0.0180 (14)	0.0033 (10)	0.0018 (10)	0.0020 (10)
C4	0.0147 (13)	0.0126 (12)	0.0118 (13)	0.0019 (10)	−0.0008 (10)	0.0000 (10)
C6	0.0120 (12)	0.0114 (12)	0.0145 (13)	0.0032 (10)	0.0009 (10)	0.0030 (10)
C3	0.0103 (12)	0.0135 (13)	0.0171 (14)	0.0009 (10)	0.0007 (10)	0.0006 (10)
C2	0.0159 (13)	0.0154 (13)	0.0140 (13)	0.0032 (11)	0.0049 (10)	0.0028 (10)
C1	0.0167 (13)	0.0149 (13)	0.0117 (13)	0.0043 (11)	0.0049 (10)	−0.0007 (10)
S1	0.0134 (3)	0.0117 (3)	0.0133 (3)	0.0020 (2)	0.0007 (2)	0.0011 (2)
O1	0.0175 (10)	0.0135 (9)	0.0128 (10)	−0.0013 (8)	0.0019 (8)	0.0007 (7)
O2	0.0141 (10)	0.0204 (10)	0.0132 (10)	0.0018 (8)	0.0024 (7)	0.0027 (8)
O3	0.0238 (11)	0.0224 (11)	0.0288 (12)	0.0118 (9)	−0.0025 (9)	0.0027 (9)
C7	0.0227 (15)	0.0121 (13)	0.0132 (13)	−0.0012 (11)	0.0032 (11)	0.0002 (10)
C8	0.0283 (17)	0.0235 (16)	0.0201 (16)	0.0042 (13)	−0.0022 (13)	−0.0014 (12)
C9	0.0343 (19)	0.0331 (19)	0.0211 (17)	−0.0046 (15)	−0.0055 (14)	0.0012 (14)
C12	0.0277 (17)	0.0256 (17)	0.0291 (18)	−0.0008 (14)	0.0098 (14)	−0.0090 (14)
C11	0.046 (2)	0.0320 (19)	0.0274 (19)	−0.0054 (17)	0.0191 (17)	−0.0105 (15)
C10	0.047 (2)	0.0265 (17)	0.0144 (16)	−0.0148 (15)	0.0065 (14)	−0.0030 (13)
C13	0.068 (3)	0.044 (2)	0.0176 (18)	−0.024 (2)	0.0062 (18)	−0.0074 (16)

### Geometric parameters (Å, °)

**Table:**

Pd1—N1	2.030 (2)	C2—C1	1.504 (4)
Pd1—N2	2.060 (2)	C2—H2B	0.9700
Pd1—Cl1	2.3053 (8)	C2—H2C	0.9700
Pd1—Cl2	2.3115 (7)	C1—H1B	0.9700
N1—C1	1.495 (3)	C1—H1C	0.9700
N1—C6	1.495 (3)	S1—O3	1.444 (2)
N1—H1A	0.82 (5)	S1—O1	1.459 (2)
N2—C3	1.493 (3)	S1—O2	1.468 (2)
N2—C2	1.504 (4)	S1—C7	1.777 (3)
N2—H2A	0.79 (5)	C7—C12	1.381 (4)
N3—C4	1.511 (4)	C7—C8	1.385 (5)
N3—C5	1.512 (4)	C8—C9	1.396 (5)
N3—H3A	0.76 (6)	C8—H8A	0.9300
N3—H3D	0.83 (6)	C9—C10	1.388 (6)
C5—C6	1.516 (4)	C9—H9A	0.9300
C5—H5A	0.9700	C12—C11	1.400 (5)
C5—H5B	0.9700	C12—H12A	0.9300
C4—C3	1.512 (4)	C11—C10	1.383 (6)
C4—H4A	0.9700	C11—H11A	0.9300
C4—H4B	0.9700	C10—C13	1.515 (5)
C6—H6A	0.9700	C13—H13A	0.9600
C6—H6B	0.9700	C13—H13B	0.9600
C3—H3B	0.9700	C13—H13C	0.9600
C3—H3C	0.9700		
			
N1—Pd1—N2	82.71 (9)	N2—C3—H3C	108.4
N1—Pd1—Cl1	92.14 (7)	C4—C3—H3C	108.4
N2—Pd1—Cl1	174.84 (7)	H3B—C3—H3C	107.4
N1—Pd1—Cl2	173.69 (7)	N2—C2—C1	109.3 (2)
N2—Pd1—Cl2	91.14 (7)	N2—C2—H2B	109.8
Cl1—Pd1—Cl2	94.02 (3)	C1—C2—H2B	109.8
C1—N1—C6	112.8 (2)	N2—C2—H2C	109.8
C1—N1—Pd1	103.19 (17)	C1—C2—H2C	109.8
C6—N1—Pd1	116.87 (17)	H2B—C2—H2C	108.3
C1—N1—H1A	108 (4)	N1—C1—C2	107.4 (2)
C6—N1—H1A	109 (4)	N1—C1—H1B	110.2
Pd1—N1—H1A	106 (4)	C2—C1—H1B	110.2
C3—N2—C2	112.6 (2)	N1—C1—H1C	110.2
C3—N2—Pd1	113.10 (17)	C2—C1—H1C	110.2
C2—N2—Pd1	109.76 (17)	H1B—C1—H1C	108.5
C3—N2—H2A	109 (4)	O3—S1—O1	113.12 (13)
C2—N2—H2A	110 (4)	O3—S1—O2	113.98 (13)
Pd1—N2—H2A	101 (4)	O1—S1—O2	111.41 (12)
C4—N3—C5	119.6 (2)	O3—S1—C7	106.63 (14)
C4—N3—H3A	108 (4)	O1—S1—C7	106.18 (13)
C5—N3—H3A	110 (4)	O2—S1—C7	104.72 (13)
C4—N3—H3D	107 (4)	C12—C7—C8	120.5 (3)
C5—N3—H3D	105 (4)	C12—C7—S1	119.9 (3)
H3A—N3—H3D	105 (5)	C8—C7—S1	119.7 (2)
N3—C5—C6	117.7 (2)	C7—C8—C9	119.3 (3)
N3—C5—H5A	107.9	C7—C8—H8A	120.3
C6—C5—H5A	107.9	C9—C8—H8A	120.3
N3—C5—H5B	107.9	C10—C9—C8	121.4 (4)
C6—C5—H5B	107.9	C10—C9—H9A	119.3
H5A—C5—H5B	107.2	C8—C9—H9A	119.3
N3—C4—C3	116.7 (2)	C7—C12—C11	119.3 (4)
N3—C4—H4A	108.1	C7—C12—H12A	120.4
C3—C4—H4A	108.1	C11—C12—H12A	120.4
N3—C4—H4B	108.1	C10—C11—C12	121.4 (3)
C3—C4—H4B	108.1	C10—C11—H11A	119.3
H4A—C4—H4B	107.3	C12—C11—H11A	119.3
N1—C6—C5	117.5 (2)	C11—C10—C9	118.1 (3)
N1—C6—H6A	107.9	C11—C10—C13	121.3 (4)
C5—C6—H6A	107.9	C9—C10—C13	120.6 (4)
N1—C6—H6B	107.9	C10—C13—H13A	109.5
C5—C6—H6B	107.9	C10—C13—H13B	109.5
H6A—C6—H6B	107.2	H13A—C13—H13B	109.5
N2—C3—C4	115.6 (2)	C10—C13—H13C	109.5
N2—C3—H3B	108.4	H13A—C13—H13C	109.5
C4—C3—H3B	108.4	H13B—C13—H13C	109.5

### Hydrogen-bond geometry (Å, °)

**Table:**

*D*—H···*A*	*D*—H	H···*A*	*D*···*A*	*D*—H···*A*
N1—H1a···O1^i^	0.82 (6)	2.05 (6)	2.833 (3)	159 (6)
N2—H2a···Cl1^ii^	0.79 (5)	2.66 (6)	3.365 (2)	149 (5)
N3—H3a···O2	0.75 (6)	2.04 (6)	2.743 (3)	156 (6)
N3—H3d···Cl2^iii^	0.84 (5)	2.31 (6)	3.136 (3)	171 (5)

Symmetry codes: (i) *x*+1, *y*+1, *z*; (ii) *x*−1, *y*, *z*;  (iii) *x*, *y*−1, *z*.

### Table 3 Bond distances and angles for hydrogen-bonding interactions (Å, °)

**Table:**

D···O	X—D···A	S=O···D
C1,C6—N1···O1^i^	2.833 (3)	97.42 (16), 104.83 (15)
N1—H1a···O1^i^	2.05 (5)	159 (5)
C4,C5—N3···O2	2.743 (3)	125.55 (18), 99.40 (17)
N3—H3a···O2	2.04 (6)	156 (6)
N3—H3d···Cl2^ii^	2.31 (6)	171 (5)
C4,C5—N3···Cl2^ii^	3.136 (3)	104.10 (16), 102.66 (16)
Pd—Cl2—H3d^iii^		85 (1)
Pd—Cl2—N3^iii^		86.58 (5)
S1—O1···N1^iv^		118.00 (12)
S1—O1···H1a^iv^		113.3 (15)
S1—O2···N3		139.70 (12)
S1—O2···H3a		133.4 (16)

Symmetry codes: (i) 1+x, 1+y, z; (ii) x, -1+y, z; (iii) x, 1+y, z; (iv) -1+x, -1+y, z.
